# A dataset for soil organic carbon in agricultural systems for the Southeast Asia region

**DOI:** 10.1038/s41597-024-03213-3

**Published:** 2024-04-12

**Authors:** Federico Gomez, Ana Carcedo, Chan Makara Mean, Manuel Reyes, Lyda Hok, Florent Tivet, Vang Seng, P. V. Vara Prasad, Ignacio Ciampitti

**Affiliations:** 1 Department of Agronomy, Kansas State University, Manhattan, Kansas USA; 2 Faculty of Agricultural Biosystems Engineering, Royal University of Agriculture, Phnom Penh, Cambodia; 3 Sustainable Intensification Innovation Lab, and Department of Agronomy, Kansas State University, Manhattan, Kansas USA; 4 Center of Excellence on Sustainable Agricultural Intensification and Nutrition, Royal University of Agriculture, Phnom Penh, Cambodia; 5 CIRAD, UPR AIDA, University of Montpellier, Montpellier, France; 6 Agroecology for South-East Asia, Vientiane, Laos; 7 Department of Agricultural Land Resources Management, Phnom Penh, Cambodia

**Keywords:** Agriculture, Agroecology

## Abstract

The determination of changes in soil organic carbon (SOC) content under different cropping systems is necessary for policy development oriented towards soil conservation, C sequestration, and future C credit markets. The aim of this study was to generate an open SOC dataset resulting from a systematic literature search related to the agricultural systems for Southeast Asia. The dataset has 209 articles and 4341 observations on soils of cropping systems in this region from articles published between 1987 and 2023. This dataset included different management practices, land uses, soil sampling depth, and length of SOC content assessment. In addition, inherent features of crop production reported in the experiments were included in the dataset. This dataset can be applied to quantify and compare the impact of different land uses or management practices on SOC content, providing foundational knowledge towards identifying sustainable practices. Lastly, it is a useful guide for future regional SOC sequestration policies and the development of C credit markets.

## Background & Summary

Soil organic carbon (SOC) is one of the main indicators of soil quality^1^. A decrease in soil quality (i.e., soil degradation) leads to crop productivity reduction^2^. Furthermore, the soil has been proposed as a sink for atmospheric C to mitigate the effects of climate change^3^. Linked to its C sink capability, the 2015 Paris Climate Conference (COP21), presented an initiative to increase soil organic matter stock by 0.4% per year in the first 30–40 cm of soil depth to compensate for the CO_2_ production derived from human activities. Achieving this goal requires a positive net SOC balance, which implies an increase in the soil C inputs, a reduction in the soil C outputs, or both processes simultaneously^4^. Finally, to strengthen current efforts on the potential for SOC sequestration, the Food and Agriculture Organization (FAO) has provided a new estimate of this potential on a global scale (Global Soil Organic Carbon Sequestration Potential Map)^5^.

On a global scale, Southeast Asia presented one of the highest rates of deforestation during the 1990s, associated with changes in land use^6^. Although deforestation in Southeast Asia continued, it occurred at a lower rate during the first decade of the 2000s^7^ and subsequent years^8^. Traditionally, the main land use in this region was shifting cultivation^9^. This production system consists of crop production (mainly rice and maize in this region) for a short period, then abandonment of the land allowing reversion to secondary forest during a long fallow period (i.e., between crops)^9,10^. Later, cropping systems were intensified, and shifting cultivation was replaced by monoculture plantations, particularly rubber plantations associated with higher profitability^10^ and annual crops^11–13^. Moreover, Indonesia has also experienced the conversion of native forests to oil palm and timber plantations between 2000 and 2016^14^, and the conversion of mangrove forests to rice crops or oil palm plantations^15^. Under these scenarios, current efforts to implement soil C conservation practices will be relevant to maintain long-term productivity of these fragile cropping systems.

A recent global meta-analysis of soil organic carbon in the Anthropocene highlighted the effect of land conversion for crop, land management, and climate change on SOC depletion^16^. The same authors identified some practices to assist in safeguarding C stocks and potentially promoting a restoration of SOC levels. Several studies in Southeast Asia have evaluated the variation in SOC as a consequence of changes in land use^17–19^, tillage system^20–22^, type of production system (i.e., organic vs. traditional)^23,24^, application of inorganic fertilizers and organic amendments or crop residues^25,26^. Understanding the impact of different management will serve as a foundational information to guide the development of new soil C conservation practices in agriculture.

However, there are few open data sources at global and regional scales compiling levels of SOC data^27,28^. The development of an open and accessible dataset would allow direct policies associated with C sequestration and to guide implementation of future market for C credits, among other potential applications. Therefore, this study presents a new dataset resulting from a systematic search of the scientific literature available related to SOC changes in agriculture for Southeast Asia region.

## Methods

### Data collection

A literature search was conducted in March 2023 in Scopus and Web of Science using as search equation in the title, abstract, or keywords: ‘Southeast Asia’ or the countries in Southeast Asia (‘Brunei’ or ‘Burma’ or ‘Myanmar’ or ‘Cambodia’ or ‘Timor-Leste’ or ‘Indonesia’ or ‘Laos’ or ‘Malaysia’ or ‘Philippines’ or ‘Singapore’ or ‘Thailand’ or ‘Vietnam’) and ‘soil organic matter’ or ‘soil organic carbon’ or ‘soil carbon’ or ‘organic carbon’ or ‘organic matter’. In Scopus, the search was restricted to the areas of Agricultural and Biological Sciences and Environmental Science and to articles and reviews written in English, and thus 2488 published articles were obtained. In Web of Science, the search was restricted to the areas of Soil Science, Environmental Sciences, Agronomy and Ecology, and to articles or reviews written in English, which resulted in 1,551 published articles. In August 2023, a bibliographic search using the equations described above was carried out in Google Scholar (using the software Publish or Perish^29^) and in Ovid. In the case of Ovid, the results were limited to articles written in English. The search in both search engines resulted in 7,448 published articles. Additionally, we added eleven articles that are part of other datasets associated with soil organic carbon^27,28^. The total number of articles obtained were 11,499.

In the first screening step, articles without DOI and duplicates were removed, keeping 4,802 articles. Then a screening by abstract was performed, in which articles that did not have information associated with soil organic carbon or soil organic matter in crop production systems were rejected, resulting in a selection of 676 articles. Screening by duplicates and by abstract was performed using the R package devtools^30^.

**Fig. 1:**
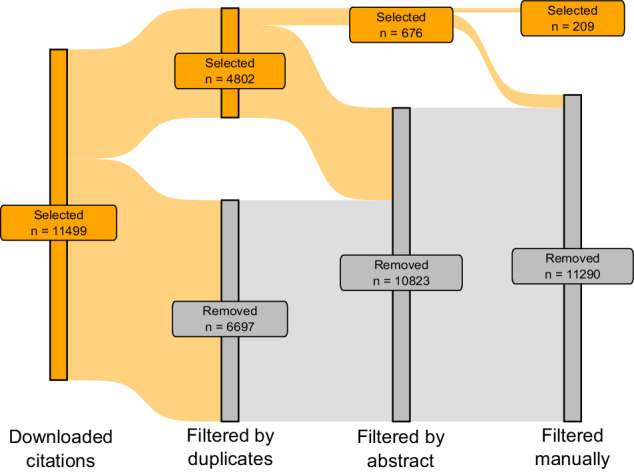
Sankey diagram showing the different screening steps employed. The ‘n’ shows the number of articles remaining in each filtering step after being selected or removed. Grey labels correspond to removed articles, while orange labels correspond to the selected articles.

Finally, a full-article manual screening was performed selecting articles which reported soil sampling depth and assessed farming practices with manipulative or observational experiments carried out under field conditions. In addition, articles were selected if treatments were applied in cropping systems or if more than two cropping systems were evaluated (i.e., data from native forests were not included). Only articles that reported measuring soil organic carbon or organic matter and the depth at which it was measured were retained. This last screening resulted in 209 selected articles with 4341 observations (Fig. 1).

The SOC, soil organic matter data (as concentration and stock), and biomass data were obtained from the articles manually, in the case of figures using WebPlotDigitizer (https://automeris.io/WebPlotDigitizer) or provided by the authors upon request. In addition, further relevant information was extracted from the articles such as the authors, year of article publication, length of the SOC assessment (i.e., the time during a treatment was applied until the SOC determination was made), implanted crop, laboratory method used for the determination of SOC, soil type, soil texture, clay content, soil pH, average annual temperature, and precipitation. We also collected data on aboveground biomass, harvestable organ yield, and residues reported in the published articles from which SOC data were extracted.

## Data Records

The data are accessible on the figshare repository^31^, available at 10.6084/m9.figshare.23736891, and which includes the following files:

1. “SOC_dataset.xlsx” includes the data. The “SOC” sheet included data collected on soil organic carbon, while the “Biomass” sheet included data collected on biomass or yield of harvestable organs of different crops.
2. “Metadata.docx” includes a summary defining each column, trait collected in the data and the units for each variable.
3. “References.docx” contains all the references of the articles included in the dataset.
4. “Code.zip” contains the scripts for the generation of the figures presented in this study.

“SOC_dataset.xlsx” contains information systematically collected from selected published articles (see Data collection section). The data presented in sheet “SOC” can be divided into three sections:

The first section referred to information inherent to the published article (id, authors, year of article publication).

The second section included information inherent to the experiment (coordinates, location, experimental design, number of replications, main descriptor, secondary descriptor, additional descriptor, length of SOC assessment). The length of the SOC assessment consists of the time of experimentation or since a treatment was applied until the SOC was determined.

The third section presented the information collected on soil organic carbon and organic matter content, and soil bulk density (values, units, and laboratory method used to determine carbon).

Finally, the crops present during the experiment, soil characteristics such as texture, soil type, soil pH, clay content, and the soil depth at which these were determined were included. Comments were added to clarify specific experimental aspects.

The data presented in sheet “Biomass” can be divided into three sections:

The first section in which information inherent to the published article was reported (id, authors, year of article publication).

The second section presented information related to the experiment (year of experimentation, number of replications, main descriptor, secondary descriptor, additional descriptor, and the crop evaluated in the experimentation).

The third section presented the information collected on aboveground biomass (crop biomass above the soil surface, excluding crop root biomass), harvestable organ yield (biomass of harvested organ in each crop), and crop residues (part of the crop aboveground biomass of a crop that is not part of the harvestable organ, this is the biomass that remains in the field after the crop is harvested) (i.e., values, units). Finally, comments were made clarifying the moisture content of the harvestable organ yield in those published articles that reported this variable.

The most relevant classification of the treatments applied in each article corresponds to main, secondary and additional descriptors.

The main descriptors are: ‘Land use’ and ‘Intervention’.

Land use corresponds to treatments in which SOC measurements were made for different cropping systems. These treatments come mainly from observational experiments.

Intervention corresponds to treatments applied in manipulative experiments related to the assessment of different management practices.

These two main descriptors were further divided into subcategories called ‘secondary descriptor’, as follows:

- Land use was divided into ‘Annual’, ‘Perennial’, and ‘Agroforestry’.
- Intervention was divided into ‘Fertilization’, ‘Tillage’, ‘Organic management’, and ‘Others’.

‘Annual’ corresponds to the cultivation of crops whose cycle is less than or equal to one year. It was normally associated with cash crops. ‘Perennial’ corresponds to the cultivation of crops whose cycle is greater than one year. It is normally associated with forestry or industrial plantations. ‘Agroforestry’ corresponds to the cultivation of annual and perennial crops combined.

‘Fertilization’ corresponds to treatments in which inorganic fertilizers or organic residues (e.g. manures, crop residues) were applied. ‘Tillage’ corresponds to treatments in which different types of soil preparation for crop implantation were carried out. ‘Organic management’ corresponds to treatments in which organic management systems (i.e., no use of synthetic chemicals) and conventional or traditional production systems were compared.

‘Others’ includes applied treatments for which there is less information, such as irrigation, burning, terracing, use of cover crops, and mulching.

Finally, the secondary descriptors were divided into sub-subcategories called ‘additional descriptor’, as follows:

- ‘Annual’ was divided into monoculture, rotation, fallow, and initial depending on whether it was a monoculture, a crop in rotation, a fallow, or an initial condition of the crop sequence to be implemented.
- ‘Perennial’ was divided into monocrop, rotation, fallow, and initial depending on whether it was a single crop, a perennial rotation crop, a fallow, or an initial condition of the crop sequence to be implemented.
- ‘Agroforestry’, kept the same name as in the secondary descriptor, to distinguish this type of cropping system from the others.
- ‘Fertilization’ was divided into with and without fertilization. In addition, the reported rate of nutrient applied was included along with this classification.
- ‘Tillage’ was divided into the tillage practices assessed in the treatments (reduced tillage, minimum tillage, conventional tillage and no-tillage). More information on the treatments applied was reported along with the tillage classification.
- ‘Organic management’ was divided into organic and non-organic management, depending on whether a non-synthetic chemical management system or a conventional system was used, respectively.
- ‘Others’ was divided into the treatments applied: pre- and postburning (for burning treatment), terraces or upland (for terrace treatment), with cover crop or mulching or without cover crop or mulching (for cover crop or mulching treatment).

**Fig. 2:**
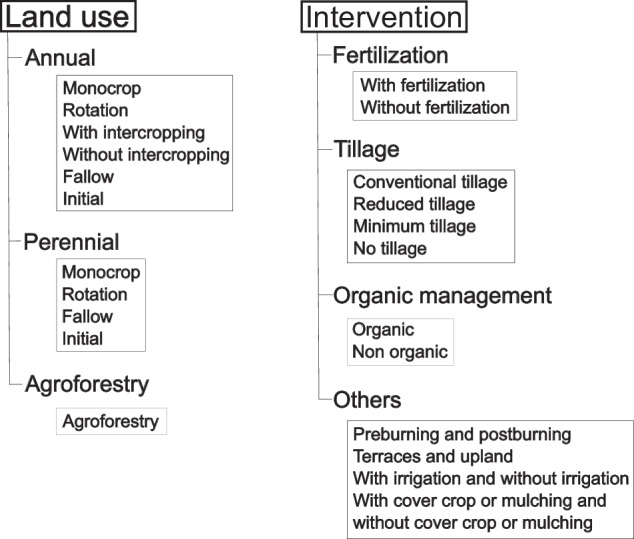
Diagram of the classification implemented for the treatments applied in each article.

This classification was summarized in Fig. 2.

### Overview of the dataset

This dataset contains information collected from papers published between 1987 and 2023. The countries with the highest number of published papers referring to SOC were Indonesia, Thailand, and Vietnam, while those countries with the lowest number of papers referring to SOC were East Timor and Myanmar. The most frequently studied crops are rice, maize, oil palm, cassava, agroforestry systems, Acacia and rubber plantations, grass, and coffee.

The most studied treatments were related to land use (63.7%). Cropping systems with annual and perennial crops are the most common (~ 88% of land-use related articles). The most common interventions were related to application of inorganic fertilizers and organic residues, implementation of different tillage systems, and implementation of organic management (i.e., no synthetic chemicals in cropping system) (~ 82%). In most published articles the length of SOC content assessment was less than 10 years.

## Technical Validation

To evaluate the reliability of the retrieved SOC dataset, a quality control was performed via visual inspection, box plots, and detection of possible outliers for the main, secondary, and additional descriptors (i.e., main annual and perennial crops for land use, and inorganic fertilizer and organic residue application for intervention).

For this purpose, the percentage of SOC was calculated for all peer-reviewed studies from the reported SOC and soil organic matter (SOM) values at different concentration units. If no coefficient of equivalence between soil organic matter and soil C (tcoef column in “SOC_dataset.xslx”) was reported, a value of 0.58 was used to estimate SOC concentration^32^. The SOC values that were considered as mild outliers were those below Q1–1.5 * IQR (lower limit) or above Q3 + 1.5 * IQR (upper limit), where Q1 and Q3 correspond to the 0.25 and 0.75 quartiles, respectively, and IQR stand for interquartile range (i.e., Q3 - Q1). For the detection of extreme outlier SOC values, the calculation was like the indicated for mild outliers except that the IQR was multiplied by 3^33^. The SOC value limits were established for the assessed descriptors (land use, and inorganic fertilizer and organic residue application). Similarly, the same process was followed for the most studied annual crops for which a greater amount of information was available (i.e., rice, maize, and cassava), and for oil palm.

**Fig. 3:**
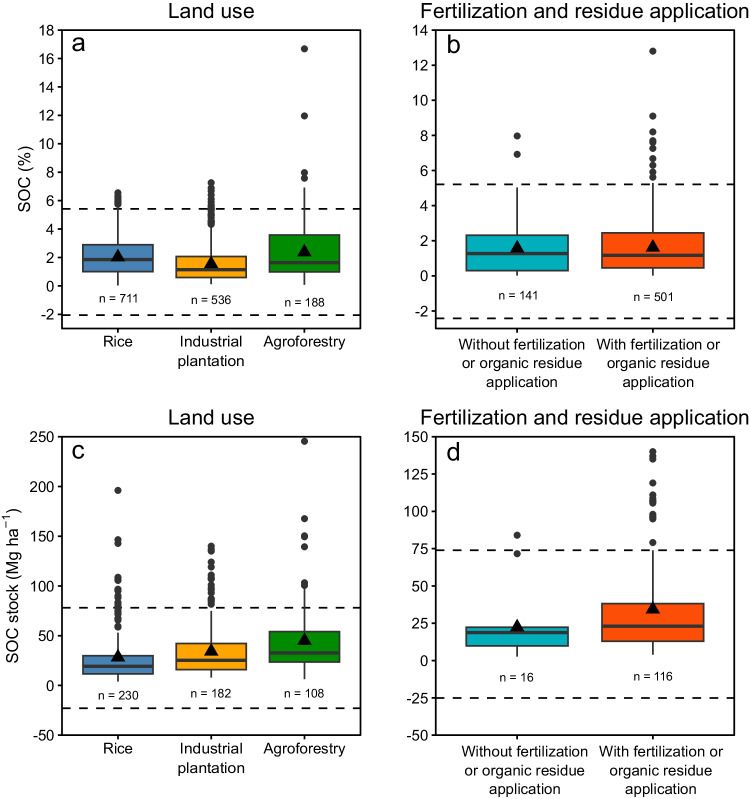
Box plot of soil organic carbon (SOC) concentration (upper panel) and SOC stock (lower panel) for the main land use (**a** and **c**), and fertilizer and organic residue application (**b** and **d**). In each main and secondary descriptor, the edges of the box correspond to the first and third quartiles (Q1 and Q3, respectively), the solid line stands for the median, the whiskers indicate the minimum and maximum value (i.e., Q1–1.5 * IQR and Q3 + 1.5 * IQR, where IQR = Q3 - Q1, the interquartile range), and the dots correspond to potential outliers. The triangles correspond to the mean SOC values for each level within treatments. The dashed horizontal lines show the lower and upper limits for mild outliers (Q1–1.5 * IQR and Q3 + 1.5 * IQR) for the treatments. ‘n’ indicates the number of observations in each additional descriptor.

Both SOC values as concentration and as stock for the main and secondary descriptors and main levels within them are presented in Fig. 3.

In the case of the main land uses, SOC values above the upper limits corresponded to peatlands (IDs 7, 36, 52, 119, 121, 125, 132, 148, 180, 181, 187, 197, which have SOC values > 40%). Peatlands are wetlands ecosystems characterized by slow organic matter decomposition, leading to its accumulation^34^. Hence, its SOC levels are very high, exceeding the levels expected in any soil used for crop production. Some SOC concentration observations were above the upper limit of mild outliers, with values between 6 and 7% (i.e., 8 observations, Fig. 3a). Only one of the remaining observations had a SOC value greater than the upper limit for extreme outliers (from ID 166, with values of 12 and 16.7%) (Fig. 3a). A few observations of SOC as stock were greater than the upper limit for mild outliers (corresponding to IDs 8, 30, 59, 61, 73, 77, 96, 100, 113, 136, 151, 152, and 200), although in any case those values did not exceed the upper limit for extreme outliers (Fig. 3b).

In fertilizer and organic residue application secondary descriptor, some observations showed SOC concentrations above the upper limit for extreme outliers (13 observations corresponding to IDs 21, 45, 145, and 209). In Fig. 3b,the SOC values corresponding to ID 66 are not shown as those exceed 40%. For SOC stock some observations were above the upper limit for mild outliers, corresponding to IDs 8, 77, and 96. (Fig. 3d).

**Fig. 4:**
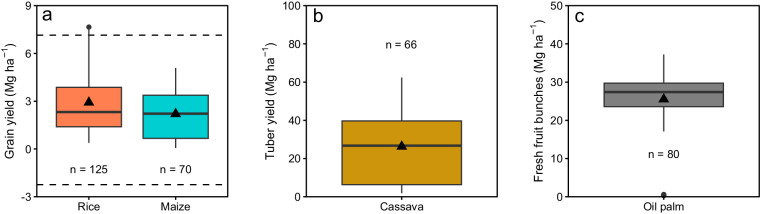
Box plots of harvestable organ yield of (**a**) maize and rice, (**b**) cassava, and (**c**) oil palm. In each crop, the edges of the box correspond to the first and third quartiles (Q1 and Q3, respectively), the solid line stands for the median, the whiskers indicate the minimum and maximum value (i.e., Q1–1.5 * IQR and Q3 + 1.5 * IQR, where IQR = Q3 - Q1, the interquartile range), and the dots correspond to potential outliers. The triangles correspond to the mean SOC values for each level within treatments. In a), the dashed horizontal lines show the lower and upper limits for mild outliers (Q1–1.5 * IQR and Q3 + 1.5 * IQR) for both crops. ‘n’ indicates the number of observations in each treatment level.

Harvestable organ yields did not exceed the limits for extreme outliers in any of the three most studied annual crops (Fig. 4a,b). A few rice grain yield values from ID 144 (i.e., n = 4) were above the upper limit of mild outliers (Fig. 4a), and oil palm fresh fruit bunch yield values from ID 154 (i.e., n = 6) were below the lower limit of extreme outliers (Fig. 4c).

## Usage Notes

The current dataset can be used to assess the overall impact of management practices (e.g., use of cover crops, fertilization, and application of organic residues) on the SOC stock under different cropping systems. Specifically, our dataset could be used to synthesize the effect of land use and management for the region and determine potential research knowledge gaps for safeguarding SOC stocks.

The dataset presented in this study will be dynamic and subject to updates. The version presented here corresponds to the peer review conducted in 2023. The main goal of this study is to create awareness of the urgent need for opening and sharing datasets to guide future critical research and policies linked to safeguarding C stocks while maintaining our projection of future food demand. Although this study focused on the Southeast Asia region, a similar approach could be implemented globally. This collaborative and interactive process will help provide a better foundation for the next generation of researchers, agronomists, farmers, and policy makers to assist with the current challenge of better food security under a more sustainable approach.

## Data Availability

The dataset and related files, and the R software scripts used to produce the figures are available on the figshare repository^31^.
